# Supplementary material for: The social dimension of apathy: evidence for a distinct domain from 11,243 individuals across health and neurocognitive disorders
Source: Transl Psychiatry. 2026 Apr 8;16:263. doi: 10.1038/s41398-026-04023-4 (PMC13184353; doi:10.1038/s41398-026-04023-4)
Supplement: Supplementary file 1 — Supplementary Material [file 41398_2026_4023_MOESM1_ESM.docx]

# Supplementary Materials

Zhao et al. 2026 “The social dimension of apathy: Evidence for a distinct domain from 11,243 individuals across health and neurocognitive disorders”. Translational Psychiatry.

#### Supplementary Table 1: Assessment mode breakdown by diagnostic group.

This table reports the number of participants assessed via online platforms versus in-person clinical or research sites, broken down by diagnostic group. Online assessments were conducted via Prolific (UK-based participants) or Credamo (China-based participants). In-person assessments were conducted at specialist clinics (Oxford, Cambridge, Canterbury, Anhui), university research sites, or schools. Group indicates the diagnostic category: HC = Healthy Controls, Depression = individuals with a formal diagnosis of Major Depressive Disorder or scoring above established cut-offs on depression screening tools, NCD = Neurocognitive Disorders (combined), with individual NCD diagnoses listed below (AD = Alzheimer's disease, MCI = Mild Cognitive Impairment, SCD = Subjective Cognitive Decline, PD = Parkinson's disease, DLB = Dementia with Lewy Bodies, FTD = Frontotemporal Dementia, AIE = Autoimmune Encephalitis, SVD = Small Vessel Disease). Online and In-Person columns report participant counts for each mode. Total is the sum of both modes. Proportion Online (%) is the percentage of participants in each group assessed online. All NCD patients were assessed exclusively in person.

| Group | Online | In-Person | Total | Proportion Online (%) |
| --- | --- | --- | --- | --- |
| Total | 6230 | 5013 | 11243 | 55.4 |
| HC | 5885 | 2880 | 8765 | 67.1 |
| Depression | 345 | 979 | 1324 | 26.1 |
| NCD | 0 | 1154 | 1154 | 0 |
| AD | 0 | 130 | 130 | 0 |
| MCI | 0 | 34 | 34 | 0 |
| SCD | 0 | 136 | 136 | 0 |
| PD | 0 | 551 | 551 | 0 |
| DLB | 0 | 39 | 39 | 0 |
| FTD | 0 | 89 | 89 | 0 |
| AIE | 0 | 86 | 86 | 0 |
| SVD | 0 | 89 | 89 | 0 |

#### Supplementary Table 2: Factor loading of all AMI, AES and DAS items.

| VariableName | Measure | Item | Factor1 | Factor2 | Factor3 | Factor4 | Factor5 |
| --- | --- | --- | --- | --- | --- | --- | --- |
| ami_3 | AMI | I enjoy doing things with people I have just met | 0.022615 | 0.087059 | 0.470036 | -0.11709 | -0.03044 |
| ami_8 | AMI | I go out with friends on a weekly basis | -0.06006 | -0.1043 | 0.822674 | -0.10704 | 0.005834 |
| aes_1 | AES | I am interested in things. | -0.06846 | 0.626662 | 0.117052 | 0.005072 | 0.175286 |
| aes_4 | AES | I am interested in having new experiences. | -0.09798 | 0.730458 | 0.144628 | -0.04469 | -0.05254 |
| aes_5 | AES | I am interested in learning new things | -0.12055 | 0.867387 | -0.09428 | -0.02962 | -0.01348 |
| aes_12 | AES | I have friends. | -0.12127 | 0.183978 | 0.693854 | -0.02724 | 0.124381 |
| aes_13 | AES | Getting together with friends is important to me. | -0.11804 | 0.107727 | 0.814611 | 0.015187 | 0.045554 |
| das_5 | DAS | I am concerned about how my family feel | 0.235757 | -0.13308 | 0.075696 | 0.409869 | -0.06864 |
| das_14 | DAS | I try new things | 0.279572 | 0.303325 | 0.20428 | -0.05169 | -0.18713 |
| das_24 | DAS | I feel indifferent to what is going on around me | -0.08317 | 0.041625 | 0.186009 | 0.316871 | 0.439922 |
| ami_4 | AMI | I suggest activities for me and my friends to do | 0.038629 | 0.044798 | 0.590646 | -0.00035 | 0.03078 |
| das_4 | DAS | I think of new things to do during the day | 0.352652 | 0.297424 | 0.078201 | -0.03231 | -0.18176 |
| ami_5 | AMI | I make decisions firmly and without hesitation | 0.419111 | 0.013753 | 0.063845 | -0.23427 | 0.052522 |
| ami_17 | AMI | I enjoy choosing what to do from a range of activities | 0.062616 | 0.347355 | 0.072575 | 0.086285 | -0.015 |
| aes_7 | AES | I approach life with intensity. | 0.259853 | 0.445839 | 0.016805 | 0.001416 | -0.10365 |
| das_8 | DAS | I plan my days activities in advance | 0.520493 | -0.12765 | 0.04413 | 0.179174 | -0.00298 |
| das_13 | DAS | I set goals for myself | 0.574001 | 0.081485 | -0.04016 | 0.074428 | -0.05929 |
| das_17 | DAS | When doing a demanding task, I have difficulty working out what I have to do | 0.095218 | 0.111098 | -0.07499 | -0.14177 | 0.549901 |
| ami_2 | AMI | I start conversations with random people | 0.11743 | -0.06268 | 0.36961 | -0.01009 | -0.09121 |
| ami_9 | AMI | When I decide to do something, I am able to make an effort easily | 0.54028 | -0.15503 | 0.225533 | -0.07572 | 0.235536 |
| ami_10 | AMI | I don't like to laze around | 0.592719 | -0.12753 | 0.031221 | -0.06747 | 0.034135 |
| ami_11 | AMI | I get things done when they need to be done, without requiring reminders from others | 0.640027 | -0.17306 | 0.005171 | 0.016291 | 0.315166 |
| ami_14 | AMI | I start conversations without being prompted | 0.082206 | 0.015403 | 0.451437 | 0.058664 | 0.059648 |
| ami_15 | AMI | When I have something I need to do, I do it straightaway so it is out of the way | 0.783389 | -0.26891 | -0.08525 | -0.02724 | 0.18936 |
| aes_2 | AES | I get things done during the day. | 0.490825 | 0.216379 | -0.07946 | 0.016603 | 0.269721 |
| aes_3 | AES | Getting things started on my own is important to me. | 0.318319 | 0.342016 | -0.1117 | -0.0542 | 0.069631 |
| aes_6 | AES | I put little effort into anything. | 0.127752 | 0.126977 | -0.09691 | 0.141168 | 0.360575 |
| aes_10 | AES | Someone has to tell me what to do each day. | 0.080953 | 0.152891 | -0.1576 | 0.089117 | 0.327242 |
| aes_16 | AES | Getting things done during the day is important to me. | 0.498339 | 0.241264 | -0.09394 | 0.147427 | 0.04793 |
| aes_17 | AES | I have initiative. | 0.461976 | 0.424351 | -0.01502 | -0.03576 | 0.045615 |
| das_1 | DAS | I need a bit of encouragement to get things started | 0.36484 | -0.01967 | -0.04304 | -0.13826 | 0.458408 |
| das_2 | DAS | I contact my friends | 0.041979 | -0.12095 | 0.803103 | -0.02828 | 0.048061 |
| das_6 | DAS | I find myself staring in to space | 0.041995 | -0.01034 | 0.028721 | -0.06821 | 0.537035 |
| das_16 | DAS | I act on things I have thought about during the day | 0.510231 | 0.083331 | -0.02517 | 0.033801 | -0.15793 |
| das_18 | DAS | I keep myself busy | 0.499714 | 0.098877 | -0.08052 | 0.096978 | 0.036078 |
| das_22 | DAS | I am spontaneous | 0.062492 | 0.277375 | 0.23262 | -0.07238 | -0.27613 |
| ami_12 | AMI | When I decide to do something, I am motivated to see it through to the end | 0.709954 | -0.12275 | -0.018 | -0.00461 | 0.219735 |
| aes_8 | AES | Seeing a job through to the end is important to me. | 0.421366 | 0.309716 | -0.20506 | 0.108946 | 0.163558 |
| aes_9 | AES | I spend time doing things that interest me. | -0.0427 | 0.6475 | -0.00674 | -0.13994 | 0.083566 |
| das_10 | DAS | I am able to focus on a task until it is finished | 0.54307 | -0.01398 | -0.11868 | 0.033454 | 0.355552 |
| das_19 | DAS | I get easily confused when doing several things at once | 0.001116 | 0.107716 | 0.165978 | -0.133 | 0.507139 |
| das_21 | DAS | I find it difficult to keep my mind on things | 0.244821 | -0.009 | 0.063477 | -0.07837 | 0.656031 |
| das_23 | DAS | I am easily distracted | 0.412838 | -0.19678 | 0.024424 | -0.10081 | 0.59796 |
| ami_1 | AMI | I feel sad or upset when I hear bad news | 0.014451 | -0.00635 | -0.06332 | 0.557456 | -0.15994 |
| ami_7 | AMI | Based on the last two weeks, I would say I care deeply about how my loved ones think of me | 0.047364 | -0.04134 | 0.11673 | 0.438543 | 0.025813 |
| ami_16 | AMI | I feel bad when I hear an acquaintance has an accident or illness | 0.013028 | 0.047459 | -0.02663 | 0.561374 | 0.030043 |
| aes_14 | AES | When something good happens, I get excited. | -0.01934 | 0.412249 | 0.226915 | 0.163938 | -0.10904 |
| das_3 | DAS | I express my emotions | 0.164648 | 0.009504 | 0.295819 | 0.243126 | 0.004815 |
| das_9 | DAS | When I receive bad news I feel bad about it | 0.040984 | 0.012172 | -0.08976 | 0.598041 | -0.13931 |
| das_12 | DAS | I struggle to empathise with other people | -0.21736 | 0.161304 | 0.102593 | 0.388551 | 0.396928 |
| das_20 | DAS | I become emotional easily when watching something happy or sad on TV | 0.091092 | -0.07168 | -0.05434 | 0.456474 | -0.23425 |
| ami_6 | AMI | After making a decision, I will wonder if I have made the wrong choice | -0.13242 | -0.05503 | -0.0564 | 0.412522 | -0.1539 |
| ami_13 | AMI | I feel awful if I say something insensitive | 0.015687 | -0.13493 | 0.013921 | 0.719404 | -0.02322 |
| ami_18 | AMI | If I realise I have been unpleasant to someone, I will feel terribly guilty afterwards | -0.05376 | 0.052597 | -0.09726 | 0.730253 | -0.02929 |
| aes_11 | AES | I am less concerned about my problems than I should be. | 0.081155 | -0.13839 | 0.064097 | 0.203454 | 0.340813 |
| das_7 | DAS | Before I do something I think about how others would feel about it | 0.126108 | -0.07166 | 0.12672 | 0.395721 | -0.05252 |
| das_15 | DAS | I am unconcerned about how others feel about my behaviour | -0.17375 | -0.053 | 0.025927 | 0.43341 | 0.24417 |
| aes_15 | AES | I have an accurate understanding of my problems. | 0.092109 | 0.314548 | 0.028452 | 0.084558 | 0.087225 |
| aes_18 | AES | I have motivation. | 0.544078 | 0.32753 | 0.020882 | -0.01976 | 0.097048 |
| das_11 | DAS | I lack motivation | 0.456626 | 0.079402 | 0.065205 | -0.06516 | 0.441757 |

#### Supplementary Figure 1: Stacked loading plot.


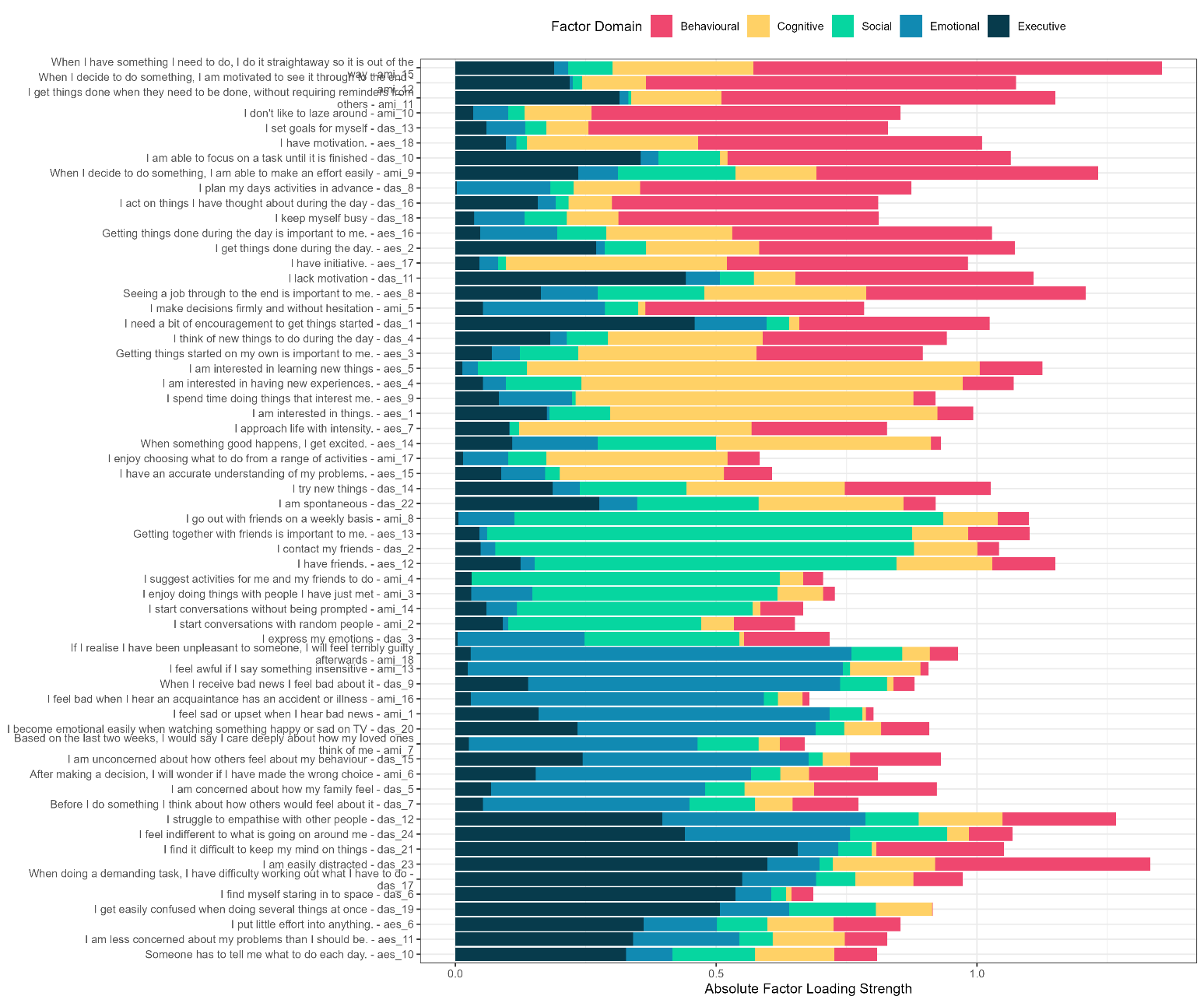


#### Supplementary Table 3: Module characteristics for the HC Cohort from 16 to 90.

This table details the properties of network modules identified across different age windows, where each row represents a single module. The columns describe the following: Age is the centre of the 10-year sliding age window (in years); ModuleID is a unique identifier for each module within that age window; CentralNode is the most influential node with the highest connection strength within its module; CentralNodeDomain is the pre-defined functional domain of that central node; ModuleSize indicates the total number of nodes in the module; DominantDomain is the most frequent functional domain among all nodes within the module; Purity measures module homogeneity as the proportion of nodes belonging to the dominant domain; and Entropy measures module diversity, with higher values indicating a greater mix of functional domains.

| Age | ModuleID | CentralNode | CentralNodeDomain | ModuleSize | DominantDomain | Purity | Entropy |
| --- | --- | --- | --- | --- | --- | --- | --- |
| 16 | 1 | ami_15 | Behavioural | 6 | Behavioural | 1 | 0 |
| 16 | 2 | ami_14 | Social | 6 | Social | 1 | 0 |
| 16 | 3 | ami_18 | Emotional | 6 | Emotional | 1 | 0 |
| 17 | 1 | ami_15 | Behavioural | 6 | Behavioural | 1 | 0 |
| 17 | 2 | ami_14 | Social | 6 | Social | 1 | 0 |
| 17 | 3 | ami_18 | Emotional | 6 | Emotional | 1 | 0 |
| 18 | 1 | ami_15 | Behavioural | 6 | Behavioural | 1 | 0 |
| 18 | 2 | ami_14 | Social | 6 | Social | 1 | 0 |
| 18 | 3 | ami_18 | Emotional | 6 | Emotional | 1 | 0 |
| 19 | 1 | ami_15 | Behavioural | 6 | Behavioural | 1 | 0 |
| 19 | 2 | ami_14 | Social | 6 | Social | 1 | 0 |
| 19 | 3 | ami_18 | Emotional | 6 | Emotional | 1 | 0 |
| 20 | 1 | ami_15 | Behavioural | 6 | Behavioural | 1 | 0 |
| 20 | 2 | ami_14 | Social | 6 | Social | 1 | 0 |
| 20 | 3 | ami_18 | Emotional | 6 | Emotional | 1 | 0 |
| 21 | 1 | ami_15 | Behavioural | 6 | Behavioural | 1 | 0 |
| 21 | 2 | ami_14 | Social | 6 | Social | 1 | 0 |
| 21 | 3 | ami_18 | Emotional | 6 | Emotional | 1 | 0 |
| 22 | 1 | ami_11 | Behavioural | 6 | Behavioural | 1 | 0 |
| 22 | 2 | ami_3 | Social | 6 | Social | 1 | 0 |
| 22 | 3 | ami_18 | Emotional | 6 | Emotional | 1 | 0 |
| 23 | 1 | ami_15 | Behavioural | 7 | Behavioural | 0.857143 | 0.591673 |
| 23 | 2 | ami_3 | Social | 6 | Social | 1 | 0 |
| 23 | 3 | ami_18 | Emotional | 5 | Emotional | 1 | 0 |
| 24 | 1 | ami_15 | Behavioural | 7 | Behavioural | 0.857143 | 0.591673 |
| 24 | 2 | ami_3 | Social | 6 | Social | 1 | 0 |
| 24 | 3 | ami_18 | Emotional | 5 | Emotional | 1 | 0 |
| 25 | 1 | ami_11 | Behavioural | 7 | Behavioural | 0.857143 | 0.591673 |
| 25 | 2 | ami_3 | Social | 6 | Social | 1 | 0 |
| 25 | 3 | ami_18 | Emotional | 5 | Emotional | 1 | 0 |
| 26 | 1 | ami_11 | Behavioural | 7 | Behavioural | 0.857143 | 0.591673 |
| 26 | 2 | ami_3 | Social | 6 | Social | 1 | 0 |
| 26 | 3 | ami_13 | Emotional | 5 | Emotional | 1 | 0 |
| 27 | 1 | ami_11 | Behavioural | 7 | Behavioural | 0.857143 | 0.591673 |
| 27 | 2 | ami_3 | Social | 6 | Social | 1 | 0 |
| 27 | 3 | ami_13 | Emotional | 5 | Emotional | 1 | 0 |
| 28 | 1 | ami_15 | Behavioural | 7 | Behavioural | 0.857143 | 0.591673 |
| 28 | 2 | ami_3 | Social | 6 | Social | 1 | 0 |
| 28 | 3 | ami_18 | Emotional | 5 | Emotional | 1 | 0 |
| 29 | 1 | ami_15 | Behavioural | 7 | Behavioural | 0.857143 | 0.591673 |
| 29 | 2 | ami_3 | Social | 6 | Social | 1 | 0 |
| 29 | 3 | ami_18 | Emotional | 5 | Emotional | 1 | 0 |
| 30 | 1 | ami_3 | Social | 6 | Social | 1 | 0 |
| 30 | 2 | ami_18 | Emotional | 5 | Emotional | 1 | 0 |
| 30 | 3 | ami_15 | Behavioural | 7 | Behavioural | 0.857143 | 0.591673 |
| 31 | 1 | ami_15 | Behavioural | 7 | Behavioural | 0.857143 | 0.591673 |
| 31 | 2 | ami_3 | Social | 6 | Social | 1 | 0 |
| 31 | 3 | ami_13 | Emotional | 5 | Emotional | 1 | 0 |
| 32 | 1 | ami_3 | Social | 6 | Social | 1 | 0 |
| 32 | 2 | ami_13 | Emotional | 5 | Emotional | 1 | 0 |
| 32 | 3 | ami_15 | Behavioural | 7 | Behavioural | 0.857143 | 0.591673 |
| 33 | 1 | ami_15 | Behavioural | 7 | Behavioural | 0.857143 | 0.591673 |
| 33 | 2 | ami_3 | Social | 6 | Social | 1 | 0 |
| 33 | 3 | ami_18 | Emotional | 5 | Emotional | 1 | 0 |
| 34 | 1 | ami_15 | Behavioural | 7 | Behavioural | 0.857143 | 0.591673 |
| 34 | 2 | ami_3 | Social | 6 | Social | 1 | 0 |
| 34 | 3 | ami_18 | Emotional | 5 | Emotional | 1 | 0 |
| 35 | 1 | ami_15 | Behavioural | 7 | Behavioural | 0.857143 | 0.591673 |
| 35 | 2 | ami_3 | Social | 6 | Social | 1 | 0 |
| 35 | 3 | ami_18 | Emotional | 5 | Emotional | 1 | 0 |
| 36 | 1 | ami_15 | Behavioural | 7 | Behavioural | 0.857143 | 0.591673 |
| 36 | 2 | ami_3 | Social | 6 | Social | 1 | 0 |
| 36 | 3 | ami_13 | Emotional | 5 | Emotional | 1 | 0 |
| 37 | 1 | ami_15 | Behavioural | 7 | Behavioural | 0.857143 | 0.591673 |
| 37 | 2 | ami_3 | Social | 6 | Social | 1 | 0 |
| 37 | 3 | ami_13 | Emotional | 5 | Emotional | 1 | 0 |
| 38 | 1 | ami_11 | Behavioural | 7 | Behavioural | 0.857143 | 0.591673 |
| 38 | 2 | ami_3 | Social | 6 | Social | 1 | 0 |
| 38 | 3 | ami_13 | Emotional | 5 | Emotional | 1 | 0 |
| 39 | 1 | ami_11 | Behavioural | 7 | Behavioural | 0.857143 | 0.591673 |
| 39 | 2 | ami_3 | Social | 6 | Social | 1 | 0 |
| 39 | 3 | ami_13 | Emotional | 5 | Emotional | 1 | 0 |
| 40 | 1 | ami_11 | Behavioural | 7 | Behavioural | 0.857143 | 0.591673 |
| 40 | 2 | ami_4 | Social | 6 | Social | 1 | 0 |
| 40 | 3 | ami_13 | Emotional | 5 | Emotional | 1 | 0 |
| 41 | 1 | ami_11 | Behavioural | 7 | Behavioural | 0.857143 | 0.591673 |
| 41 | 2 | ami_4 | Social | 6 | Social | 1 | 0 |
| 41 | 3 | ami_13 | Emotional | 5 | Emotional | 1 | 0 |
| 42 | 1 | ami_11 | Behavioural | 7 | Behavioural | 0.857143 | 0.591673 |
| 42 | 2 | ami_4 | Social | 6 | Social | 1 | 0 |
| 42 | 3 | ami_13 | Emotional | 5 | Emotional | 1 | 0 |
| 43 | 1 | ami_11 | Behavioural | 7 | Behavioural | 0.857143 | 0.591673 |
| 43 | 2 | ami_4 | Social | 6 | Social | 1 | 0 |
| 43 | 3 | ami_13 | Emotional | 5 | Emotional | 1 | 0 |
| 44 | 1 | ami_11 | Behavioural | 7 | Behavioural | 0.857143 | 0.591673 |
| 44 | 2 | ami_4 | Social | 6 | Social | 1 | 0 |
| 44 | 3 | ami_13 | Emotional | 5 | Emotional | 1 | 0 |
| 45 | 1 | ami_11 | Behavioural | 7 | Behavioural | 0.857143 | 0.591673 |
| 45 | 2 | ami_4 | Social | 6 | Social | 1 | 0 |
| 45 | 3 | ami_18 | Emotional | 5 | Emotional | 1 | 0 |
| 46 | 1 | ami_11 | Behavioural | 7 | Behavioural | 0.857143 | 0.591673 |
| 46 | 2 | ami_3 | Social | 6 | Social | 1 | 0 |
| 46 | 3 | ami_18 | Emotional | 5 | Emotional | 1 | 0 |
| 47 | 1 | ami_11 | Behavioural | 7 | Behavioural | 0.857143 | 0.591673 |
| 47 | 2 | ami_3 | Social | 6 | Social | 1 | 0 |
| 47 | 3 | ami_18 | Emotional | 5 | Emotional | 1 | 0 |
| 48 | 1 | ami_12 | Behavioural | 7 | Behavioural | 0.857143 | 0.591673 |
| 48 | 2 | ami_3 | Social | 6 | Social | 1 | 0 |
| 48 | 3 | ami_18 | Emotional | 5 | Emotional | 1 | 0 |
| 49 | 1 | ami_12 | Behavioural | 7 | Behavioural | 0.857143 | 0.591673 |
| 49 | 2 | ami_2 | Social | 3 | Social | 1 | 0 |
| 49 | 3 | ami_4 | Social | 3 | Social | 1 | 0 |
| 49 | 4 | ami_18 | Emotional | 5 | Emotional | 1 | 0 |
| 50 | 1 | ami_12 | Behavioural | 7 | Behavioural | 0.857143 | 0.591673 |
| 50 | 2 | ami_2 | Social | 3 | Social | 1 | 0 |
| 50 | 3 | ami_4 | Social | 3 | Social | 1 | 0 |
| 50 | 4 | ami_18 | Emotional | 5 | Emotional | 1 | 0 |
| 51 | 1 | ami_12 | Behavioural | 7 | Behavioural | 0.857143 | 0.591673 |
| 51 | 2 | ami_2 | Social | 3 | Social | 1 | 0 |
| 51 | 3 | ami_4 | Social | 3 | Social | 1 | 0 |
| 51 | 4 | ami_18 | Emotional | 5 | Emotional | 1 | 0 |
| 52 | 1 | ami_3 | Social | 6 | Social | 1 | 0 |
| 52 | 2 | ami_13 | Emotional | 5 | Emotional | 1 | 0 |
| 52 | 3 | ami_12 | Behavioural | 7 | Behavioural | 0.857143 | 0.591673 |
| 53 | 1 | ami_12 | Behavioural | 7 | Behavioural | 0.857143 | 0.591673 |
| 53 | 2 | ami_3 | Social | 6 | Social | 1 | 0 |
| 53 | 3 | ami_13 | Emotional | 5 | Emotional | 1 | 0 |
| 54 | 1 | ami_3 | Social | 6 | Social | 1 | 0 |
| 54 | 2 | ami_13 | Emotional | 5 | Emotional | 1 | 0 |
| 54 | 3 | ami_12 | Behavioural | 7 | Behavioural | 0.857143 | 0.591673 |
| 55 | 1 | ami_12 | Behavioural | 7 | Behavioural | 0.857143 | 0.591673 |
| 55 | 2 | ami_3 | Social | 6 | Social | 1 | 0 |
| 55 | 3 | ami_13 | Emotional | 5 | Emotional | 1 | 0 |
| 56 | 1 | ami_12 | Behavioural | 7 | Behavioural | 0.857143 | 0.591673 |
| 56 | 2 | ami_4 | Social | 6 | Social | 1 | 0 |
| 56 | 3 | ami_13 | Emotional | 5 | Emotional | 1 | 0 |
| 57 | 1 | ami_12 | Behavioural | 7 | Behavioural | 0.857143 | 0.591673 |
| 57 | 2 | ami_4 | Social | 6 | Social | 1 | 0 |
| 57 | 3 | ami_13 | Emotional | 5 | Emotional | 1 | 0 |
| 58 | 1 | ami_12 | Behavioural | 6 | Behavioural | 1 | 0 |
| 58 | 2 | ami_4 | Social | 6 | Social | 1 | 0 |
| 58 | 3 | ami_13 | Emotional | 6 | Emotional | 1 | 0 |
| 59 | 1 | ami_12 | Behavioural | 6 | Behavioural | 1 | 0 |
| 59 | 2 | ami_4 | Social | 6 | Social | 1 | 0 |
| 59 | 3 | ami_13 | Emotional | 6 | Emotional | 1 | 0 |
| 60 | 1 | ami_12 | Behavioural | 6 | Behavioural | 1 | 0 |
| 60 | 2 | ami_4 | Social | 6 | Social | 1 | 0 |
| 60 | 3 | ami_13 | Emotional | 6 | Emotional | 1 | 0 |
| 61 | 1 | ami_12 | Behavioural | 7 | Behavioural | 0.857143 | 0.591673 |
| 61 | 2 | ami_4 | Social | 5 | Social | 1 | 0 |
| 61 | 3 | ami_13 | Emotional | 6 | Emotional | 1 | 0 |
| 62 | 1 | ami_12 | Behavioural | 8 | Behavioural | 0.75 | 0.811278 |
| 62 | 2 | ami_3 | Social | 2 | Social | 1 | 0 |
| 62 | 3 | ami_4 | Social | 2 | Social | 1 | 0 |
| 62 | 4 | ami_13 | Emotional | 6 | Emotional | 1 | 0 |
| 63 | 1 | ami_12 | Behavioural | 8 | Behavioural | 0.75 | 0.811278 |
| 63 | 2 | ami_3 | Social | 2 | Social | 1 | 0 |
| 63 | 3 | ami_4 | Social | 2 | Social | 1 | 0 |
| 63 | 4 | ami_13 | Emotional | 6 | Emotional | 1 | 0 |
| 64 | 1 | ami_12 | Behavioural | 8 | Behavioural | 0.75 | 0.811278 |
| 64 | 2 | ami_3 | Social | 2 | Social | 1 | 0 |
| 64 | 3 | ami_4 | Social | 2 | Social | 1 | 0 |
| 64 | 4 | ami_13 | Emotional | 6 | Emotional | 1 | 0 |
| 65 | 1 | ami_12 | Behavioural | 8 | Behavioural | 0.75 | 0.811278 |
| 65 | 2 | ami_3 | Social | 2 | Social | 1 | 0 |
| 65 | 3 | ami_4 | Social | 2 | Social | 1 | 0 |
| 65 | 4 | ami_18 | Emotional | 6 | Emotional | 1 | 0 |
| 66 | 1 | ami_12 | Behavioural | 8 | Behavioural | 0.75 | 0.811278 |
| 66 | 2 | ami_3 | Social | 2 | Social | 1 | 0 |
| 66 | 3 | ami_4 | Social | 2 | Social | 1 | 0 |
| 66 | 4 | ami_18 | Emotional | 6 | Emotional | 1 | 0 |
| 67 | 1 | ami_12 | Behavioural | 8 | Behavioural | 0.75 | 0.811278 |
| 67 | 2 | ami_3 | Social | 2 | Social | 1 | 0 |
| 67 | 3 | ami_4 | Social | 2 | Social | 1 | 0 |
| 67 | 4 | ami_18 | Emotional | 6 | Emotional | 1 | 0 |
| 68 | 1 | ami_12 | Behavioural | 8 | Behavioural | 0.75 | 0.811278 |
| 68 | 2 | ami_3 | Social | 2 | Social | 1 | 0 |
| 68 | 3 | ami_4 | Social | 2 | Social | 1 | 0 |
| 68 | 4 | ami_18 | Emotional | 6 | Emotional | 1 | 0 |
| 69 | 1 | ami_12 | Behavioural | 8 | Behavioural | 0.75 | 0.811278 |
| 69 | 2 | ami_3 | Social | 2 | Social | 1 | 0 |
| 69 | 3 | ami_4 | Social | 2 | Social | 1 | 0 |
| 69 | 4 | ami_18 | Emotional | 6 | Emotional | 1 | 0 |
| 70 | 1 | ami_12 | Behavioural | 8 | Behavioural | 0.75 | 0.811278 |
| 70 | 2 | ami_3 | Social | 2 | Social | 1 | 0 |
| 70 | 3 | ami_4 | Social | 2 | Social | 1 | 0 |
| 70 | 4 | ami_18 | Emotional | 6 | Emotional | 1 | 0 |
| 71 | 1 | ami_12 | Behavioural | 7 | Behavioural | 0.857143 | 0.591673 |
| 71 | 2 | ami_3 | Social | 2 | Social | 1 | 0 |
| 71 | 3 | ami_4 | Social | 3 | Social | 1 | 0 |
| 71 | 4 | ami_18 | Emotional | 6 | Emotional | 1 | 0 |
| 72 | 1 | ami_12 | Behavioural | 8 | Behavioural | 0.75 | 0.811278 |
| 72 | 2 | ami_3 | Social | 2 | Social | 1 | 0 |
| 72 | 3 | ami_4 | Social | 2 | Social | 1 | 0 |
| 72 | 4 | ami_18 | Emotional | 6 | Emotional | 1 | 0 |
| 73 | 1 | ami_12 | Behavioural | 8 | Behavioural | 0.75 | 0.811278 |
| 73 | 2 | ami_3 | Social | 2 | Social | 1 | 0 |
| 73 | 3 | ami_4 | Social | 2 | Social | 1 | 0 |
| 73 | 4 | ami_18 | Emotional | 6 | Emotional | 1 | 0 |
| 74 | 1 | ami_12 | Behavioural | 8 | Behavioural | 0.75 | 0.811278 |
| 74 | 2 | ami_4 | Social | 4 | Social | 1 | 0 |
| 74 | 3 | ami_18 | Emotional | 6 | Emotional | 1 | 0 |
| 75 | 1 | ami_11 | Behavioural | 8 | Behavioural | 0.75 | 0.811278 |
| 75 | 2 | ami_4 | Social | 6 | Social | 0.666667 | 0.918296 |
| 75 | 3 | ami_18 | Emotional | 4 | Emotional | 1 | 0 |
| 76 | 1 | ami_12 | Behavioural | 8 | Behavioural | 0.75 | 0.811278 |
| 76 | 2 | ami_4 | Social | 6 | Social | 0.666667 | 0.918296 |
| 76 | 3 | ami_18 | Emotional | 4 | Emotional | 1 | 0 |
| 77 | 1 | ami_12 | Behavioural | 9 | Behavioural | 0.666667 | 1.224394 |
| 77 | 2 | ami_4 | Social | 6 | Social | 0.666667 | 0.918296 |
| 77 | 3 | ami_13 | Emotional | 3 | Emotional | 1 | 0 |
| 78 | 1 | ami_12 | Behavioural | 9 | Behavioural | 0.666667 | 1.224394 |
| 78 | 2 | ami_4 | Social | 6 | Social | 0.666667 | 0.918296 |
| 78 | 3 | ami_13 | Emotional | 3 | Emotional | 1 | 0 |
| 79 | 1 | ami_12 | Behavioural | 8 | Behavioural | 0.75 | 0.811278 |
| 79 | 2 | ami_4 | Social | 6 | Social | 0.666667 | 0.918296 |
| 79 | 3 | ami_16 | Emotional | 4 | Emotional | 1 | 0 |
| 80 | 1 | ami_12 | Behavioural | 10 | Behavioural | 0.6 | 1.370951 |
| 80 | 2 | ami_4 | Social | 5 | Social | 0.8 | 0.721928 |
| 80 | 3 | ami_13 | Emotional | 3 | Emotional | 1 | 0 |
| 81 | 1 | ami_11 | Behavioural | 9 | Behavioural | 0.666667 | 1.224394 |
| 81 | 2 | ami_16 | Emotional | 4 | Emotional | 1 | 0 |
| 81 | 3 | ami_4 | Social | 5 | Social | 0.8 | 0.721928 |
| 82 | 1 | ami_12 | Behavioural | 10 | Behavioural | 0.6 | 1.370951 |
| 82 | 2 | ami_2 | Social | 4 | Social | 1 | 0 |
| 82 | 3 | ami_18 | Emotional | 4 | Emotional | 1 | 0 |
| 83 | 1 | ami_9 | Behavioural | 6 | Behavioural | 0.666667 | 1.251629 |
| 83 | 2 | ami_2 | Social | 4 | Social | 1 | 0 |
| 83 | 3 | ami_12 | Behavioural | 8 | Emotional | 0.625 | 1.298795 |
| 84 | 1 | ami_2 | Social | 7 | Social | 0.714286 | 1.148835 |
| 84 | 2 | ami_12 | Behavioural | 11 | Behavioural | 0.454545 | 1.348588 |
| 85 | 1 | ami_9 | Behavioural | 3 | Behavioural | 0.666667 | 0.918296 |
| 85 | 2 | ami_2 | Social | 6 | Social | 0.833333 | 0.650022 |
| 85 | 3 | ami_12 | Behavioural | 9 | Emotional | 0.555556 | 1.351644 |

#### Supplementary Table 4: Module characteristics for the Depression cohort.

| Age | ModuleID | CentralNode | CentralNodeDomain | ModuleSize | DominantDomain | Purity | Entropy |
| --- | --- | --- | --- | --- | --- | --- | --- |
| 16 | 1 | ami_12 | Behavioural | 6 | Behavioural | 1 | 0 |
| 16 | 2 | ami_2 | Social | 5 | Social | 1 | 0 |
| 16 | 3 | ami_18 | Emotional | 7 | Emotional | 0.857143 | 0.591673 |
| 17 | 1 | ami_12 | Behavioural | 6 | Behavioural | 1 | 0 |
| 17 | 2 | ami_2 | Social | 5 | Social | 1 | 0 |
| 17 | 3 | ami_18 | Emotional | 7 | Emotional | 0.857143 | 0.591673 |
| 18 | 1 | ami_12 | Behavioural | 6 | Behavioural | 1 | 0 |
| 18 | 2 | ami_2 | Social | 5 | Social | 1 | 0 |
| 18 | 3 | ami_18 | Emotional | 7 | Emotional | 0.857143 | 0.591673 |
| 19 | 1 | ami_12 | Behavioural | 6 | Behavioural | 1 | 0 |
| 19 | 2 | ami_3 | Social | 6 | Social | 1 | 0 |
| 19 | 3 | ami_13 | Emotional | 6 | Emotional | 1 | 0 |
| 20 | 1 | ami_12 | Behavioural | 6 | Behavioural | 1 | 0 |
| 20 | 2 | ami_2 | Social | 3 | Social | 1 | 0 |
| 20 | 3 | ami_4 | Social | 3 | Social | 1 | 0 |
| 20 | 4 | ami_13 | Emotional | 6 | Emotional | 1 | 0 |
| 21 | 1 | ami_12 | Behavioural | 6 | Behavioural | 1 | 0 |
| 21 | 2 | ami_2 | Social | 3 | Social | 1 | 0 |
| 21 | 3 | ami_4 | Social | 3 | Social | 1 | 0 |
| 21 | 4 | ami_18 | Emotional | 6 | Emotional | 1 | 0 |
| 22 | 1 | ami_12 | Behavioural | 5 | Behavioural | 1 | 0 |
| 22 | 2 | ami_3 | Social | 4 | Social | 0.75 | 0.811278 |
| 22 | 3 | ami_4 | Social | 3 | Social | 1 | 0 |
| 22 | 4 | ami_18 | Emotional | 6 | Emotional | 1 | 0 |
| 23 | 1 | ami_12 | Behavioural | 6 | Behavioural | 0.833333 | 0.650022 |
| 23 | 2 | ami_5 | Behavioural | 2 | Behavioural | 0.5 | 1 |
| 23 | 3 | ami_3 | Social | 2 | Social | 1 | 0 |
| 23 | 4 | ami_4 | Social | 3 | Social | 1 | 0 |
| 23 | 5 | ami_13 | Emotional | 5 | Emotional | 1 | 0 |
| 24 | 1 | ami_12 | Behavioural | 5 | Behavioural | 1 | 0 |
| 24 | 2 | ami_5 | Behavioural | 3 | Behavioural | 0.333333 | 1.584963 |
| 24 | 3 | ami_3 | Social | 2 | Social | 1 | 0 |
| 24 | 4 | ami_4 | Social | 3 | Social | 1 | 0 |
| 24 | 5 | ami_13 | Emotional | 5 | Emotional | 1 | 0 |
| 25 | 1 | ami_12 | Behavioural | 5 | Behavioural | 1 | 0 |
| 25 | 2 | ami_14 | Social | 8 | Social | 0.75 | 1.061278 |
| 25 | 3 | ami_13 | Emotional | 5 | Emotional | 1 | 0 |
| 26 | 1 | ami_12 | Behavioural | 5 | Behavioural | 1 | 0 |
| 26 | 2 | ami_14 | Social | 8 | Social | 0.75 | 1.061278 |
| 26 | 3 | ami_13 | Emotional | 5 | Emotional | 1 | 0 |
| 27 | 1 | ami_12 | Behavioural | 5 | Behavioural | 1 | 0 |
| 27 | 2 | ami_4 | Social | 8 | Social | 0.75 | 1.061278 |
| 27 | 3 | ami_13 | Emotional | 5 | Emotional | 1 | 0 |
| 28 | 1 | ami_4 | Social | 5 | Social | 1 | 0 |
| 28 | 2 | ami_13 | Emotional | 5 | Emotional | 1 | 0 |
| 28 | 3 | ami_12 | Behavioural | 8 | Behavioural | 0.75 | 1.061278 |
| 29 | 1 | ami_12 | Behavioural | 8 | Behavioural | 0.75 | 1.061278 |
| 29 | 2 | ami_4 | Social | 5 | Social | 1 | 0 |
| 29 | 3 | ami_13 | Emotional | 5 | Emotional | 1 | 0 |
| 30 | 1 | ami_12 | Behavioural | 8 | Behavioural | 0.75 | 1.061278 |
| 30 | 2 | ami_4 | Social | 5 | Social | 1 | 0 |
| 30 | 3 | ami_13 | Emotional | 5 | Emotional | 1 | 0 |
| 31 | 1 | ami_12 | Behavioural | 8 | Behavioural | 0.75 | 1.061278 |
| 31 | 2 | ami_4 | Social | 5 | Social | 1 | 0 |
| 31 | 3 | ami_13 | Emotional | 5 | Emotional | 1 | 0 |
| 32 | 1 | ami_12 | Behavioural | 8 | Behavioural | 0.75 | 1.061278 |
| 32 | 2 | ami_4 | Social | 5 | Social | 1 | 0 |
| 32 | 3 | ami_13 | Emotional | 5 | Emotional | 1 | 0 |
| 33 | 1 | ami_12 | Behavioural | 8 | Behavioural | 0.75 | 1.061278 |
| 33 | 2 | ami_4 | Social | 5 | Social | 1 | 0 |
| 33 | 3 | ami_13 | Emotional | 5 | Emotional | 1 | 0 |
| 34 | 1 | ami_15 | Behavioural | 3 | Behavioural | 1 | 0 |
| 34 | 2 | ami_4 | Social | 5 | Social | 1 | 0 |
| 34 | 3 | ami_13 | Emotional | 5 | Emotional | 1 | 0 |
| 34 | 4 | ami_5 | Behavioural | 5 | Behavioural | 0.6 | 1.370951 |
| 35 | 1 | ami_15 | Behavioural | 3 | Behavioural | 1 | 0 |
| 35 | 2 | ami_5 | Behavioural | 5 | Behavioural | 0.6 | 1.370951 |
| 35 | 3 | ami_4 | Social | 5 | Social | 1 | 0 |
| 35 | 4 | ami_13 | Emotional | 5 | Emotional | 1 | 0 |
| 36 | 1 | ami_15 | Behavioural | 3 | Behavioural | 1 | 0 |
| 36 | 2 | ami_5 | Behavioural | 5 | Behavioural | 0.6 | 1.370951 |
| 36 | 3 | ami_4 | Social | 5 | Social | 1 | 0 |
| 36 | 4 | ami_18 | Emotional | 5 | Emotional | 1 | 0 |
| 37 | 1 | ami_15 | Behavioural | 3 | Behavioural | 1 | 0 |
| 37 | 2 | ami_5 | Behavioural | 5 | Behavioural | 0.6 | 1.370951 |
| 37 | 3 | ami_4 | Social | 5 | Social | 1 | 0 |
| 37 | 4 | ami_18 | Emotional | 5 | Emotional | 1 | 0 |
| 38 | 1 | ami_15 | Behavioural | 3 | Behavioural | 1 | 0 |
| 38 | 2 | ami_5 | Behavioural | 5 | Behavioural | 0.6 | 1.370951 |
| 38 | 3 | ami_4 | Social | 5 | Social | 1 | 0 |
| 38 | 4 | ami_18 | Emotional | 5 | Emotional | 1 | 0 |
| 39 | 1 | ami_15 | Behavioural | 3 | Behavioural | 1 | 0 |
| 39 | 2 | ami_5 | Behavioural | 5 | Behavioural | 0.6 | 1.370951 |
| 39 | 3 | ami_4 | Social | 5 | Social | 1 | 0 |
| 39 | 4 | ami_18 | Emotional | 5 | Emotional | 1 | 0 |
| 40 | 1 | ami_15 | Behavioural | 3 | Behavioural | 1 | 0 |
| 40 | 2 | ami_5 | Behavioural | 5 | Behavioural | 0.6 | 1.370951 |
| 40 | 3 | ami_2 | Social | 3 | Social | 1 | 0 |
| 40 | 4 | ami_4 | Social | 2 | Social | 1 | 0 |
| 40 | 5 | ami_18 | Emotional | 3 | Emotional | 1 | 0 |
| 40 | 6 | ami_16 | Emotional | 2 | Emotional | 1 | 0 |
| 41 | 1 | ami_15 | Behavioural | 3 | Behavioural | 1 | 0 |
| 41 | 2 | ami_9 | Behavioural | 5 | Behavioural | 0.6 | 1.370951 |
| 41 | 3 | ami_4 | Social | 5 | Social | 1 | 0 |
| 41 | 4 | ami_18 | Emotional | 5 | Emotional | 1 | 0 |
| 42 | 1 | ami_15 | Behavioural | 5 | Behavioural | 1 | 0 |
| 42 | 2 | ami_5 | Behavioural | 2 | Behavioural | 0.5 | 1 |
| 42 | 3 | ami_2 | Social | 3 | Social | 1 | 0 |
| 42 | 4 | ami_4 | Social | 3 | Social | 1 | 0 |
| 42 | 5 | ami_13 | Emotional | 5 | Emotional | 1 | 0 |
| 43 | 1 | ami_15 | Behavioural | 3 | Behavioural | 1 | 0 |
| 43 | 2 | ami_2 | Social | 3 | Social | 1 | 0 |
| 43 | 3 | ami_4 | Social | 3 | Social | 1 | 0 |
| 43 | 4 | ami_13 | Emotional | 5 | Emotional | 1 | 0 |
| 43 | 5 | ami_9 | Behavioural | 4 | Behavioural | 0.75 | 0.811278 |
| 44 | 1 | ami_15 | Behavioural | 5 | Behavioural | 1 | 0 |
| 44 | 2 | ami_5 | Behavioural | 2 | Behavioural | 0.5 | 1 |
| 44 | 3 | ami_2 | Social | 3 | Social | 1 | 0 |
| 44 | 4 | ami_4 | Social | 3 | Social | 1 | 0 |
| 44 | 5 | ami_18 | Emotional | 5 | Emotional | 1 | 0 |
| 45 | 1 | ami_15 | Behavioural | 5 | Behavioural | 1 | 0 |
| 45 | 2 | ami_2 | Social | 3 | Social | 1 | 0 |
| 45 | 3 | ami_4 | Social | 3 | Social | 1 | 0 |
| 45 | 4 | ami_18 | Emotional | 5 | Emotional | 1 | 0 |
| 45 | 5 | ami_5 | Behavioural | 2 | Behavioural | 0.5 | 1 |
| 46 | 1 | ami_15 | Behavioural | 6 | Behavioural | 0.666667 | 0.918296 |
| 46 | 2 | ami_2 | Social | 4 | Social | 1 | 0 |
| 46 | 3 | ami_16 | Emotional | 6 | Emotional | 0.833333 | 0.650022 |
| 46 | 4 | ami_5 | Behavioural | 2 | Behavioural | 0.5 | 1 |
| 47 | 1 | ami_15 | Behavioural | 5 | Behavioural | 1 | 0 |
| 47 | 2 | ami_2 | Social | 7 | Social | 0.857143 | 0.591673 |
| 47 | 3 | ami_13 | Emotional | 6 | Emotional | 1 | 0 |
| 48 | 1 | ami_15 | Behavioural | 6 | Behavioural | 0.833333 | 0.650022 |
| 48 | 2 | ami_2 | Social | 5 | Social | 0.8 | 0.721928 |
| 48 | 3 | ami_13 | Emotional | 7 | Emotional | 0.857143 | 0.591673 |
| 49 | 1 | ami_12 | Behavioural | 8 | Behavioural | 0.625 | 1.298795 |
| 49 | 2 | ami_2 | Social | 4 | Social | 1 | 0 |
| 49 | 3 | ami_18 | Emotional | 2 | Emotional | 1 | 0 |
| 49 | 4 | ami_16 | Emotional | 2 | Emotional | 1 | 0 |
| 49 | 5 | ami_5 | Behavioural | 2 | Behavioural | 0.5 | 1 |
| 50 | 1 | ami_12 | Behavioural | 6 | Behavioural | 0.833333 | 0.650022 |
| 50 | 2 | ami_3 | Social | 4 | Social | 1 | 0 |
| 50 | 3 | ami_13 | Emotional | 6 | Emotional | 0.833333 | 0.650022 |
| 50 | 4 | ami_5 | Behavioural | 2 | Behavioural | 0.5 | 1 |
| 51 | 1 | ami_12 | Behavioural | 6 | Behavioural | 0.833333 | 0.650022 |
| 51 | 2 | ami_5 | Behavioural | 2 | Behavioural | 0.5 | 1 |
| 51 | 3 | ami_2 | Social | 4 | Social | 1 | 0 |
| 51 | 4 | ami_13 | Emotional | 6 | Emotional | 0.833333 | 0.650022 |
| 52 | 1 | ami_12 | Behavioural | 5 | Behavioural | 1 | 0 |
| 52 | 2 | ami_2 | Social | 5 | Social | 0.6 | 1.370951 |
| 52 | 3 | ami_4 | Social | 3 | Social | 1 | 0 |
| 52 | 4 | ami_18 | Emotional | 5 | Emotional | 1 | 0 |
| 53 | 1 | ami_12 | Behavioural | 5 | Behavioural | 1 | 0 |
| 53 | 2 | ami_2 | Social | 5 | Social | 0.6 | 1.370951 |
| 53 | 3 | ami_4 | Social | 3 | Social | 1 | 0 |
| 53 | 4 | ami_18 | Emotional | 5 | Emotional | 1 | 0 |
| 54 | 1 | ami_12 | Behavioural | 5 | Behavioural | 1 | 0 |
| 54 | 2 | ami_2 | Social | 5 | Social | 0.6 | 1.370951 |
| 54 | 3 | ami_4 | Social | 3 | Social | 1 | 0 |
| 54 | 4 | ami_18 | Emotional | 5 | Emotional | 1 | 0 |
| 55 | 1 | ami_12 | Behavioural | 5 | Behavioural | 1 | 0 |
| 55 | 2 | ami_5 | Behavioural | 2 | Behavioural | 0.5 | 1 |
| 55 | 3 | ami_4 | Social | 6 | Social | 1 | 0 |
| 55 | 4 | ami_18 | Emotional | 5 | Emotional | 1 | 0 |
| 56 | 1 | ami_12 | Behavioural | 6 | Behavioural | 0.833333 | 0.650022 |
| 56 | 2 | ami_5 | Behavioural | 2 | Behavioural | 0.5 | 1 |
| 56 | 3 | ami_4 | Social | 5 | Social | 1 | 0 |
| 56 | 4 | ami_18 | Emotional | 5 | Emotional | 1 | 0 |
| 57 | 1 | ami_15 | Behavioural | 5 | Behavioural | 1 | 0 |
| 57 | 2 | ami_3 | Social | 2 | Social | 1 | 0 |
| 57 | 3 | ami_4 | Social | 3 | Social | 1 | 0 |
| 57 | 4 | ami_18 | Emotional | 5 | Emotional | 1 | 0 |
| 57 | 5 | ami_5 | Behavioural | 3 | Behavioural | 0.333333 | 1.584963 |
| 58 | 1 | ami_15 | Behavioural | 7 | Behavioural | 0.857143 | 0.591673 |
| 58 | 2 | ami_3 | Social | 2 | Social | 1 | 0 |
| 58 | 3 | ami_4 | Social | 3 | Social | 1 | 0 |
| 58 | 4 | ami_18 | Emotional | 6 | Emotional | 1 | 0 |
| 59 | 1 | ami_12 | Behavioural | 7 | Behavioural | 0.857143 | 0.591673 |
| 59 | 2 | ami_4 | Social | 5 | Social | 1 | 0 |
| 59 | 3 | ami_18 | Emotional | 6 | Emotional | 1 | 0 |
| 60 | 1 | ami_15 | Behavioural | 8 | Behavioural | 0.75 | 1.061278 |
| 60 | 2 | ami_4 | Social | 5 | Social | 1 | 0 |
| 60 | 3 | ami_18 | Emotional | 5 | Emotional | 1 | 0 |
| 61 | 1 | ami_11 | Behavioural | 8 | Behavioural | 0.75 | 1.061278 |
| 61 | 2 | ami_3 | Social | 2 | Social | 1 | 0 |
| 61 | 3 | ami_8 | Social | 3 | Social | 0.666667 | 0.918296 |
| 61 | 4 | ami_18 | Emotional | 5 | Emotional | 0.8 | 0.721928 |
| 62 | 1 | ami_11 | Behavioural | 8 | Behavioural | 0.75 | 1.061278 |
| 62 | 2 | ami_4 | Social | 5 | Social | 0.8 | 0.721928 |
| 62 | 3 | ami_18 | Emotional | 5 | Emotional | 0.8 | 0.721928 |
| 63 | 1 | ami_11 | Behavioural | 7 | Behavioural | 0.714286 | 1.148835 |
| 63 | 2 | ami_3 | Social | 2 | Social | 1 | 0 |
| 63 | 3 | ami_4 | Social | 4 | Social | 0.5 | 1.5 |
| 63 | 4 | ami_18 | Emotional | 5 | Emotional | 0.8 | 0.721928 |
| 64 | 1 | ami_10 | Behavioural | 5 | Behavioural | 0.8 | 0.721928 |
| 64 | 2 | ami_3 | Social | 2 | Social | 1 | 0 |
| 64 | 3 | ami_9 | Behavioural | 5 | Behavioural | 0.4 | 1.521928 |
| 64 | 4 | ami_16 | Emotional | 6 | Emotional | 0.666667 | 0.918296 |
| 65 | 1 | ami_11 | Behavioural | 7 | Behavioural | 0.857143 | 0.591673 |
| 65 | 2 | ami_3 | Social | 3 | Social | 0.666667 | 0.918296 |
| 65 | 3 | ami_8 | Social | 3 | Social | 1 | 0 |
| 65 | 4 | ami_13 | Emotional | 5 | Emotional | 0.8 | 0.721928 |

#### Supplementary Table 5: Module characteristics for the Neurological Conditions cohort.

| Age | ModuleID | CentralNode | CentralNodeDomain | ModuleSize | DominantDomain | Purity | Entropy |
| --- | --- | --- | --- | --- | --- | --- | --- |
| 43 | 1 | ami_11 | Behavioural | 6 | Behavioural | 0.83333333 | 0.65002242 |
| 43 | 2 | ami_2 | Social | 3 | Social | 1 | 0 |
| 43 | 3 | ami_18 | Emotional | 5 | Emotional | 0.8 | 0.72192809 |
| 43 | 4 | ami_12 | Behavioural | 4 | Emotional | 0.5 | 1.5 |
| 44 | 1 | ami_12 | Behavioural | 7 | Emotional | 0.57142857 | 1.37878349 |
| 44 | 2 | ami_15 | Behavioural | 7 | Behavioural | 0.71428571 | 1.14883485 |
| 44 | 3 | ami_2 | Social | 4 | Social | 0.75 | 0.81127812 |
| 45 | 1 | ami_15 | Behavioural | 7 | Behavioural | 0.71428571 | 1.14883485 |
| 45 | 2 | ami_2 | Social | 4 | Social | 0.75 | 0.81127812 |
| 45 | 3 | ami_12 | Behavioural | 7 | Emotional | 0.57142857 | 1.37878349 |
| 46 | 1 | ami_15 | Behavioural | 7 | Behavioural | 0.71428571 | 1.14883485 |
| 46 | 2 | ami_2 | Social | 4 | Social | 1 | 0 |
| 46 | 3 | ami_16 | Emotional | 7 | Emotional | 0.71428571 | 1.14883485 |
| 47 | 1 | ami_15 | Behavioural | 8 | Behavioural | 0.75 | 1.06127812 |
| 47 | 2 | ami_14 | Social | 5 | Social | 1 | 0 |
| 47 | 3 | ami_18 | Emotional | 5 | Emotional | 1 | 0 |
| 48 | 1 | ami_15 | Behavioural | 7 | Behavioural | 0.85714286 | 0.59167278 |
| 48 | 2 | ami_3 | Social | 6 | Social | 1 | 0 |
| 48 | 3 | ami_18 | Emotional | 5 | Emotional | 1 | 0 |
| 49 | 1 | ami_9 | Behavioural | 8 | Behavioural | 0.75 | 1.06127812 |
| 49 | 2 | ami_3 | Social | 5 | Social | 1 | 0 |
| 49 | 3 | ami_18 | Emotional | 5 | Emotional | 1 | 0 |
| 50 | 1 | ami_12 | Behavioural | 7 | Behavioural | 0.85714286 | 0.59167278 |
| 50 | 2 | ami_3 | Social | 6 | Social | 1 | 0 |
| 50 | 3 | ami_18 | Emotional | 5 | Emotional | 1 | 0 |
| 51 | 1 | ami_12 | Behavioural | 7 | Behavioural | 0.85714286 | 0.59167278 |
| 51 | 2 | ami_3 | Social | 6 | Social | 1 | 0 |
| 51 | 3 | ami_18 | Emotional | 5 | Emotional | 1 | 0 |
| 52 | 1 | ami_12 | Behavioural | 5 | Behavioural | 1 | 0 |
| 52 | 2 | ami_3 | Social | 4 | Social | 0.75 | 0.81127812 |
| 52 | 3 | ami_4 | Social | 3 | Social | 1 | 0 |
| 52 | 4 | ami_18 | Emotional | 6 | Emotional | 1 | 0 |
| 53 | 1 | ami_12 | Behavioural | 7 | Behavioural | 0.71428571 | 0.86312057 |
| 53 | 2 | ami_3 | Social | 5 | Social | 0.8 | 0.72192809 |
| 53 | 3 | ami_18 | Emotional | 6 | Emotional | 1 | 0 |
| 54 | 1 | ami_12 | Behavioural | 7 | Behavioural | 0.71428571 | 0.86312057 |
| 54 | 2 | ami_3 | Social | 5 | Social | 0.8 | 0.72192809 |
| 54 | 3 | ami_18 | Emotional | 6 | Emotional | 1 | 0 |
| 55 | 1 | ami_12 | Behavioural | 6 | Behavioural | 0.83333333 | 0.65002242 |
| 55 | 2 | ami_4 | Social | 6 | Social | 0.83333333 | 0.65002242 |
| 55 | 3 | ami_18 | Emotional | 6 | Emotional | 1 | 0 |
| 56 | 1 | ami_12 | Behavioural | 6 | Behavioural | 0.83333333 | 0.65002242 |
| 56 | 2 | ami_4 | Social | 6 | Social | 0.83333333 | 0.65002242 |
| 56 | 3 | ami_18 | Emotional | 6 | Emotional | 1 | 0 |
| 57 | 1 | ami_12 | Behavioural | 6 | Behavioural | 0.83333333 | 0.65002242 |
| 57 | 2 | ami_4 | Social | 6 | Social | 0.83333333 | 0.65002242 |
| 57 | 3 | ami_18 | Emotional | 6 | Emotional | 1 | 0 |
| 58 | 1 | ami_12 | Behavioural | 6 | Behavioural | 0.83333333 | 0.65002242 |
| 58 | 2 | ami_4 | Social | 6 | Social | 0.83333333 | 0.65002242 |
| 58 | 3 | ami_18 | Emotional | 6 | Emotional | 1 | 0 |
| 59 | 1 | ami_12 | Behavioural | 6 | Behavioural | 0.83333333 | 0.65002242 |
| 59 | 2 | ami_4 | Social | 6 | Social | 0.83333333 | 0.65002242 |
| 59 | 3 | ami_18 | Emotional | 6 | Emotional | 1 | 0 |
| 60 | 1 | ami_12 | Behavioural | 9 | Behavioural | 0.66666667 | 1.22439445 |
| 60 | 2 | ami_4 | Social | 4 | Social | 1 | 0 |
| 60 | 3 | ami_18 | Emotional | 5 | Emotional | 1 | 0 |
| 61 | 1 | ami_12 | Behavioural | 9 | Behavioural | 0.66666667 | 1.22439445 |
| 61 | 2 | ami_4 | Social | 4 | Social | 1 | 0 |
| 61 | 3 | ami_18 | Emotional | 5 | Emotional | 1 | 0 |
| 62 | 1 | ami_12 | Behavioural | 9 | Behavioural | 0.66666667 | 1.22439445 |
| 62 | 2 | ami_4 | Social | 4 | Social | 1 | 0 |
| 62 | 3 | ami_18 | Emotional | 5 | Emotional | 1 | 0 |
| 63 | 1 | ami_12 | Behavioural | 8 | Behavioural | 0.75 | 1.06127812 |
| 63 | 2 | ami_3 | Social | 5 | Social | 1 | 0 |
| 63 | 3 | ami_18 | Emotional | 5 | Emotional | 1 | 0 |
| 64 | 1 | ami_12 | Behavioural | 7 | Behavioural | 0.71428571 | 1.14883485 |
| 64 | 2 | ami_4 | Social | 6 | Social | 0.83333333 | 0.65002242 |
| 64 | 3 | ami_18 | Emotional | 5 | Emotional | 1 | 0 |
| 65 | 1 | ami_12 | Behavioural | 7 | Behavioural | 0.85714286 | 0.59167278 |
| 65 | 2 | ami_4 | Social | 4 | Social | 1 | 0 |
| 65 | 3 | ami_16 | Emotional | 7 | Emotional | 0.71428571 | 0.86312057 |
| 66 | 1 | ami_12 | Behavioural | 7 | Behavioural | 0.85714286 | 0.59167278 |
| 66 | 2 | ami_4 | Social | 4 | Social | 1 | 0 |
| 66 | 3 | ami_16 | Emotional | 7 | Emotional | 0.71428571 | 0.86312057 |
| 67 | 1 | ami_12 | Behavioural | 7 | Behavioural | 0.85714286 | 0.59167278 |
| 67 | 2 | ami_4 | Social | 4 | Social | 1 | 0 |
| 67 | 3 | ami_16 | Emotional | 7 | Emotional | 0.71428571 | 0.86312057 |
| 68 | 1 | ami_12 | Behavioural | 7 | Behavioural | 0.85714286 | 0.59167278 |
| 68 | 2 | ami_4 | Social | 4 | Social | 1 | 0 |
| 68 | 3 | ami_16 | Emotional | 7 | Emotional | 0.71428571 | 0.86312057 |
| 69 | 1 | ami_12 | Behavioural | 7 | Behavioural | 0.85714286 | 0.59167278 |
| 69 | 2 | ami_4 | Social | 4 | Social | 1 | 0 |
| 69 | 3 | ami_16 | Emotional | 7 | Emotional | 0.71428571 | 0.86312057 |
| 70 | 1 | ami_12 | Behavioural | 7 | Behavioural | 0.85714286 | 0.59167278 |
| 70 | 2 | ami_4 | Social | 4 | Social | 1 | 0 |
| 70 | 3 | ami_16 | Emotional | 7 | Emotional | 0.71428571 | 0.86312057 |
| 71 | 1 | ami_12 | Behavioural | 7 | Behavioural | 0.85714286 | 0.59167278 |
| 71 | 2 | ami_4 | Social | 4 | Social | 1 | 0 |
| 71 | 3 | ami_16 | Emotional | 7 | Emotional | 0.71428571 | 0.86312057 |
| 72 | 1 | ami_12 | Behavioural | 7 | Behavioural | 0.85714286 | 0.59167278 |
| 72 | 2 | ami_4 | Social | 4 | Social | 1 | 0 |
| 72 | 3 | ami_16 | Emotional | 7 | Emotional | 0.71428571 | 0.86312057 |
| 73 | 1 | ami_12 | Behavioural | 7 | Behavioural | 0.85714286 | 0.59167278 |
| 73 | 2 | ami_4 | Social | 4 | Social | 1 | 0 |
| 73 | 3 | ami_16 | Emotional | 7 | Emotional | 0.71428571 | 0.86312057 |
| 74 | 1 | ami_4 | Social | 4 | Social | 1 | 0 |
| 74 | 2 | ami_16 | Emotional | 7 | Emotional | 0.71428571 | 0.86312057 |
| 74 | 3 | ami_12 | Behavioural | 7 | Behavioural | 0.85714286 | 0.59167278 |
| 75 | 1 | ami_12 | Behavioural | 6 | Behavioural | 1 | 0 |
| 75 | 2 | ami_4 | Social | 5 | Social | 0.8 | 0.72192809 |
| 75 | 3 | ami_18 | Emotional | 7 | Emotional | 0.71428571 | 0.86312057 |
| 76 | 1 | ami_12 | Behavioural | 6 | Behavioural | 1 | 0 |
| 76 | 2 | ami_3 | Social | 4 | Social | 1 | 0 |
| 76 | 3 | ami_18 | Emotional | 8 | Emotional | 0.75 | 0.81127812 |
| 77 | 1 | ami_12 | Behavioural | 6 | Behavioural | 1 | 0 |
| 77 | 2 | ami_3 | Social | 6 | Social | 1 | 0 |
| 77 | 3 | ami_18 | Emotional | 6 | Emotional | 1 | 0 |
| 78 | 1 | ami_12 | Behavioural | 6 | Behavioural | 1 | 0 |
| 78 | 2 | ami_3 | Social | 6 | Social | 1 | 0 |
| 78 | 3 | ami_18 | Emotional | 6 | Emotional | 1 | 0 |
| 79 | 1 | ami_12 | Behavioural | 7 | Behavioural | 0.85714286 | 0.59167278 |
| 79 | 2 | ami_3 | Social | 6 | Social | 1 | 0 |
| 79 | 3 | ami_18 | Emotional | 5 | Emotional | 1 | 0 |
| 80 | 1 | ami_12 | Behavioural | 7 | Behavioural | 0.85714286 | 0.59167278 |
| 80 | 2 | ami_3 | Social | 6 | Social | 1 | 0 |
| 80 | 3 | ami_16 | Emotional | 5 | Emotional | 1 | 0 |
| 81 | 1 | ami_12 | Behavioural | 7 | Behavioural | 0.85714286 | 0.59167278 |
| 81 | 2 | ami_3 | Social | 6 | Social | 1 | 0 |
| 81 | 3 | ami_16 | Emotional | 5 | Emotional | 1 | 0 |
| 82 | 1 | ami_12 | Behavioural | 7 | Behavioural | 0.85714286 | 0.59167278 |
| 82 | 2 | ami_3 | Social | 6 | Social | 1 | 0 |
| 82 | 3 | ami_16 | Emotional | 5 | Emotional | 1 | 0 |
| 83 | 1 | ami_11 | Behavioural | 4 | Behavioural | 0.75 | 0.81127812 |
| 83 | 2 | ami_3 | Social | 6 | Social | 0.83333333 | 0.65002242 |
| 83 | 3 | ami_10 | Behavioural | 2 | Behavioural | 0.5 | 1 |
| 83 | 4 | ami_16 | Emotional | 6 | Emotional | 0.83333333 | 0.65002242 |
| 84 | 1 | ami_11 | Behavioural | 3 | Behavioural | 1 | 0 |
| 84 | 2 | ami_10 | Behavioural | 2 | Behavioural | 0.5 | 1 |
| 84 | 3 | ami_3 | Social | 7 | Social | 0.71428571 | 1.14883485 |
| 84 | 4 | ami_16 | Emotional | 6 | Emotional | 0.83333333 | 0.65002242 |
| 85 | 1 | ami_11 | Behavioural | 3 | Behavioural | 1 | 0 |
| 85 | 2 | ami_10 | Behavioural | 2 | Behavioural | 0.5 | 1 |
| 85 | 3 | ami_3 | Social | 7 | Social | 0.71428571 | 1.14883485 |
| 85 | 4 | ami_16 | Emotional | 6 | Emotional | 0.83333333 | 0.65002242 |
| 86 | 1 | ami_16 | Emotional | 8 | Emotional | 0.625 | 0.954434 |
| 86 | 2 | ami_14 | Social | 3 | Behavioural | 0.66666667 | 0.91829583 |
| 86 | 3 | ami_3 | Social | 7 | Social | 0.71428571 | 1.14883485 |
| 87 | 1 | ami_12 | Behavioural | 9 | Emotional | 0.55555556 | 0.99107606 |
| 87 | 2 | ami_5 | Behavioural | 2 | Behavioural | 0.5 | 1 |
| 87 | 3 | ami_3 | Social | 7 | Social | 0.71428571 | 1.14883485 |
| 88 | 1 | ami_3 | Social | 7 | Social | 0.71428571 | 1.14883485 |
| 88 | 2 | ami_12 | Behavioural | 11 | Behavioural | 0.45454545 | 1.3485879 |
| 89 | 1 | ami_3 | Social | 7 | Social | 0.71428571 | 1.14883485 |
| 89 | 2 | ami_12 | Behavioural | 11 | Behavioural | 0.45454545 | 1.3485879 |

#### Supplementary Table 6: Sensitivity analysis of network module structure by assessment mode in healthy controls.

This table reports the results of a sensitivity analysis in which the Healthy Control (HC) cohort was split by assessment mode (online vs. in-person) and the Louvain community detection algorithm was applied independently to each subgroup (1,000 iterations per subgroup). Each row describes one network module. Subgroup indicates the assessment mode (HC Online: participants assessed via Prolific or Credamo, n=5885; HC InPerson: participants assessed at university or clinical research sites, n=2880). N is the number of participants in the subgroup. Module is the module identifier within each subgroup's partition. ModuleSize is the number of AMI items assigned to that module. DominantDomain is the most frequent a priori AMI domain (Behavioural, Social, or Emotional) among the items in the module. Purity is the proportion of items belonging to the dominant domain (range 0-1; 1.0 = all items from a single domain). N_Behavioural, N_Social, and N_Emotional report the number of items from each domain within the module. Stability is the proportion of 1,000 Louvain iterations that converged on the most frequent partition (range 0-1). Both subgroups yielded three modules corresponding to Behavioural, Social, and Emotional domains, with the Social module achieving 100% purity in both cases.

| Subgroup | Module | Module size | Dominant domain | Purity | N_Behavioural | N_Social | N_Emotional | Stability |
| --- | --- | --- | --- | --- | --- | --- | --- | --- |
| HC  Online | 1 | 7 | Behavioural | 0.86 | 6 | 0 | 1 | 1 |
|  | 2 | 6 | Social | 1 | 0 | 6 | 0 | 1 |
|  | 3 | 5 | Emotional | 1 | 0 | 0 | 5 | 1 |
| HC  InPerson | 1 | 6 | Behavioural | 1 | 6 | 0 | 0 | 1 |
|  | 2 | 6 | Social | 1 | 0 | 6 | 0 | 1 |
|  | 3 | 6 | Emotional | 1 | 0 | 0 | 6 | 1 |
